# Supplementary material for: Ubiquitin-Specific Protease 6 n-Terminal-like Protein (USP6NL) and the Epidermal Growth Factor Receptor (EGFR) Signaling Axis Regulates Ubiquitin-Mediated DNA Repair and Temozolomide-Resistance in Glioblastoma
Source: Biomedicines. 2022 Jun 28;10(7):1531. doi: 10.3390/biomedicines10071531 (PMC9312792; doi:10.3390/biomedicines10071531)
Supplement: Supplementary file 1 [file biomedicines-10-01531-s001.zip › biomedicines-1738502-SI.pdf]

SUPPLEMENTARY INFORMATION

Ubiquitin-specific protease 6 n-terminal-like protein (USP6NL) and epidermal growth factor receptor (EGFR) signaling axis regu-lates the ubiquitin-mediated DNA repair and temozolomide-resistance in glioblastoma

I-Chang Su[1,2,3,4], Yu-Kai Su[1,2,3,4], Hao-Yu Chuang[5,6,7], Vijesh Kumar Yadav[7], Syahru Agung Setiawan[8], Iat-Hang Fong[1,2,3,4], Chi-Tai Yeh[1,2,3,4,9*], Hui-Chuan Huang[10] and Chien-Min Lin[1,2,3,4*]

1 Graduate Institute of Clinical Medicine, College of Medicine, Taipei Medical University, Taipei City 11031, Taiwan.

2 Department of Neurology, School of Medicine, College of Medicine, Taipei Medical University, Taipei City 11031, Taiwan.

3 Division of Neurosurgery, Department of Surgery, Taipei Medical University-Shuang Ho Hospital, New Taipei City 23561, Taiwan.

4 Taipei Neuroscience Institute, Taipei Medical University, Taipei 11031, Taiwan.

5 Cell Therapy Center, Tainan Municipal An-Nan Hospital-China Medical University, Tainan, Taiwan.

6 Division of Neurosurgery, Tainan Municipal An-Nan Hospital-China Medical University, Tainan, Taiwan.

7 Division of Neurosurgery, China Medical University Beigang Hospital, Yunlin, Taiwan.

8 International Ph.D. Program in Medicine, College of Medicine, Taipei Medical University, Taipei City 11031, Taiwan

9 Department of Medical Research & Education, Shuang Ho Hospital, Taipei Medical University, New Taipei City 23561, Taiwan.

10 College of Nursing, Taipei Medical University, Taipei, Taiwan.

*Corresponding author

Dr. Chien-Min Lin, Division of Neurosurgery, Department of Surgery, Taipei Medical University-Shuang Ho Hospital, New Taipei City 23561, Taiwan. E-mail: m513092004@tmu.edu.tw

Dr. Chi-Tai Yeh, Department of Medical Research & Education, Shuang Ho Hospital, Taipei Medical University, New Taipei City 23561, Taiwan. E-mail: ctyeh@s.tmu.edu.tw

Supplementary Table

Supplementary Table S1. Baseline clinical characteristics and outcome of GBM patients in SHH-GBM cohort.

**Characteristics (n=60)**		**Cases**	**Percentage (%)**
**Age**	Less than or equal 60 y.o	34	52.3%
	More than 60 y.o	31	47.7%
**Gender**	Male	32	49.2%
	Female	33	50.8%
**Tumor Location**	Frontal	17	26.2%
	Parietal	6	9.2%
	Temporal	21	32.3%
	Occipital	1	1.5%
	Others	20	30.8%
**Recurrences**	Yes	3	4.6%
	No	62	95.4%

y.o: years old

Supplementary Table S2. List of primary antibodies used for immunoblotting and immunostaining.

Primary antibody	Company	Host species	Clonality	Concentration	Catalogue Number
**USP6NL**	Creative Diagnostics	Rabbit	Monoclonal	1:1,000 (WB)<br>1:100 (IHC)	DCABH-17363
**EGFR**	Abcam	Rabbit	Monoclonal	1:1,000 (WB)<br>1:20 (IP)	ab52894
**CD44**	Cell Signaling	Mouse	Monoclonal	1:1,000 (WB)	3570
**ABCG2**	Cell Signaling	Rabbit	Monoclonal	1:5,000 (WB)	3579
**Nanog**	Cell Signaling	Rabbit	Monoclonal	1:5,000 (WB)	4903
**CD133**	Proteintech	Rabbit	Polyclonal	1:1,000 (WB)	18495-1-AP
**SOX2**	GeneTex	Mouse	Monoclonal	1:1,000 (WB)	GTX627405
**E-cadherin**	Cell Signaling	Rabbit	Monoclonal	1:100 (IF)	3195
**N-cadherin**	Cell Signaling	Rabbit	Monoclonal	1:100 (IF)	13116
**γH2AX**	Cell Signaling	Rabbit	Monoclonal	1:100 (IF)	7631
**RAD51**	Abcam	Rabbit	Polyclonal	1:100 (IF)	ab63801
**BRCA2**	ThermoFisher	Rabbit	Polyclonal	1:100 (IF)	PA5-96128
**ATG5**	Abcam	Rabbit	Polyclonal	1:100 (IF)<br>1:2,000 (WB)	ab228668
**ATG7**	Abcam	Rabbit	Monoclonal	1:100 (IF)<br>1:2,000 (WB)	ab133528
**LC3A/B**	Cell Signaling	Rabbit	Polyclonal	1:100 (IF)<br>1:1,000 (WB)	4108
**Beclin-1**	Abcam	Rabbit	Polyclonal	1:100 (IF)<br>1:2,000 (WB)	ab62557
**GAPDH**	PROTEINTECH	Mouse	Monoclonal	1:10,000 (WB)	60004-1-Ig

WB: western blot; IF: immunofluorescence; IHC: immunohistochemistry; IP: immunoprecipitation

Supplementary Table S3. Cohen's Kappa values for each pathologist, assessing expression Q-score of USP6L in glioma tissue.

	Pathologist_A				
**Pathologist_B**	**0-75**	**76-150**	**151-225**	**226-300**	
**0-75**	2	0	0	0	2 (6.7%)
**76-150**	1	8	0	0	9 (30.0%)
**151-225**	0	5	5	1	11 (36.7%)
**226-300**	0	0	0	8	8 (26.7%)
	3 (10.0%)	13 (43.3%)	5 (16.7%)	9 (30.0%)	30

**Weighted Kappa**[a]	0.78170
**Standard error**	0.07644
**95% CI**	0.63188 to 0.93153
**P-value**	1.07e-09

[a] Linear weights

Supplementary Table S4. List of primers used for qRT-PCR amplification of each gene.

Gene	Strand	Sequence of Primers
**USP6NL**	Forward	5′-TACTCAGCCTTTCAACTC-3′
	Reverse	5′-GCAAGTACACGTCAAATC-3
**CD44**	Forward	5'-CAACACAAATGGCTGGTACG-3′
	Reverse	5'-GTGTGGTTGAAATGGTGCTG-3′
**ABCG2**	Forward	5'-TTTCAGCCGTGGAACTCTTT-3′
	Reverse	5'-TGAGTCCTGGGCAGAAGTTT-3′
**Nanog**	Forward	5′-GTGATTTGTGGGCCTGAAGA-3′
	Reverse	5′-ACACAGCTGGGTGGAAGAGA-3′
**CD133**	Forward	5′-TCTTGACCGACTGAGACCCAAC-3′
	Reverse	5′-ACTTGATGGATGCACCAAGCAC-3′
**SOX2**	Forward	5′-GACAGTTACGCGCACATGAA-3′
	Reverse	5′-TAGGTCTGCGAGCTGGTCAT-3′
**β-actin**	Forward	5′-CCCTAAGGCCAACCGTGAA-3′
	Reverse	5′-CCAGAGGCATACAGGGACAAC-3′

Supplementary Figure

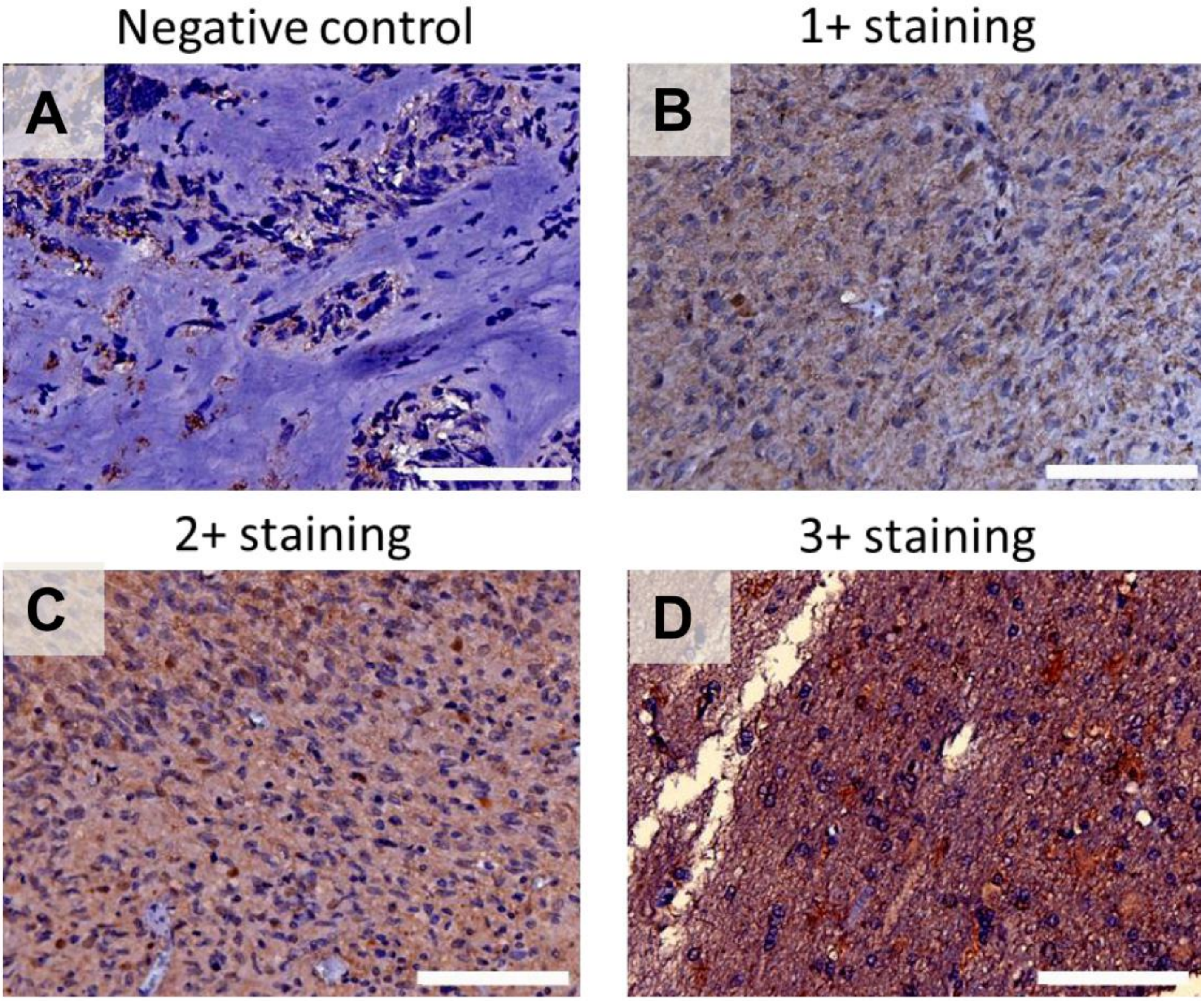

Supplementary Figure S1. Representative staining of USP6L and control in glioma FFPE tissue sections. Panel (A) is demonstrating a representative picture of negative control (glioma tissue stained with isotype rabbit IgG antibody), panel (B) is a representative picture of a 1+ positive glioma sample, panel (C) a representative picture of a 2+ positive glioma sample, and panel (D) a representative picture of a 3+ positive or the highest intensity of USP6L expression in glioma sample. Scale bar = 200 μm.
